# Lectures on Materia Medica and Therapeutics, Delivered in the College of Physicians and Surgeons of the University of the State of New York

**Published:** 1857-02

**Authors:** 


*Lectures on Materia Medica and Therapeutics, delivered in the College of Physicians and Surgeons of the University of the State of New York.* By John B. Beck, M. D., Late Professor of Materia Medica and Medical Jurisprudence. Prepared for the press by his friend C. R. Gilman, M. D., Professor of Obstetrics, etc., in the College of Physicians and Surgeons, N. Y. Second edition. New York: S. S. & W. Wood. 1856.

We are glad to see a second edition of this valuable work. New York has few names more worthy than those of the Becks. John, and Lewis, and Romeyn Beck, have each in their several departments made valuable additions to our medical literature, and now that under the auspices of Prof. Gilman, and for the benefit of the widow, this volume is thus posthumously issued, we are sure that it will be cordially received by the profession. We cannot, perhaps, give any criticism which more foreshadows the character of these lectures, than the following paragraph from Prof. Gilman's preface:

"He was in truth, as these lectures in almost every page will prove, not a runner after new things; his study was much more into the indications of treatment, the circumstances modifying the operation of medicine, and those kindred topics which I should call the philosophy of Materia Medica, than into the character and claims of new and fashionable therapeutic agents. This explains the fact that many 'new things' found no place in his lectures. I had no disposition to alter them; in this respect I shared his opinions, and concurred heartily in his plan of teaching."

Prof. Gilman makes these remarks as an apology—none was needed—for his additions. We quote them, however, as indicative of the fact that the practitioner will find in this volume the body of assured truth, and not vain attempts at novelty. He will find a strong spirit of criticism throughout, an investigating mind, one disposed rather to winnow out than to multiply our cumbersome materia medica. He will find, too, no tendency to heroism in practice, no fondness for the *nimia diligentia.*

As a book which stands by the old land-marks, and is conservative without stupidity, we commend this volume to the intelligent practitioner as one full of information, and possessed of reliability and honesty. The profession owes something to Prof. Gilman for this labor of love. His own light is buried under the bushel of too much modesty, or, at any rate, too small an estimate of his own powers. Occasionally we hear enough from him to lead us to wish for more, but he is remarkably continent.

For sale by Phinney & Co.

